# Urinary N-Acetyl-Beta-D-Glucosaminidase levels predict immunoglobulin a nephropathy remission status

**DOI:** 10.1186/s12882-023-03262-7

**Published:** 2023-07-14

**Authors:** Xiao Liu, Shaomin Gong, Yichun Ning, Yang Li, Huili Zhou, Luna He, Lin Lin, Shi Jin, Ziyan Shen, Bowen Zhu, Fang Li, Jie Li, Xiao Tan, Xiaoyan Jiao, Yiqin Shi, Xiaoqiang Ding

**Affiliations:** Department of Nephrology, Zhongshan Hospital, Fudan University, No.180, Fenglin Road, Xuhui District, Shanghai, 200032 China

**Keywords:** Biomarker, IgA nephropathy, Urinary N-acetyl-beta-D-glucosaminidase, Serum cystatin C, Remission status

## Abstract

**Background:**

Tubulointerstitial lesions play a pivotal role in the progression of IgA nephropathy (IgAN). Elevated N-acetyl-beta-D-glucosaminidase (NAG) in urine is released from damaged proximal tubular epithelial cells (PTEC) and may serve as a biomarker of renal progression in diseases with tubulointerstitial involvement.

**Methods:**

We evaluated the predictive value of urinary NAG (uNAG) for disease progression in 213 biopsy-proven primary IgAN patients from January 2018 to December 2019 at Zhongshan Hospital, Fudan University. We compared the results with those of serum cystatin C (sCysC).

**Results:**

Increased uNAG and sCysC levels were associated with worse clinical and histological manifestations. Only uNAG level was independently associated with remission status after adjustment. Patients with high uNAG levels (> 22.32 U/g Cr) had a 4.32-fold greater risk of disease progression. The combination of baseline uNAG and clinical data may achieve satisfactory risk prediction in IgAN patients with relatively preserved renal function (eGFR ≥ 60 ml/min/1.73 m^2^, area under the curve [AUC] 0.760).

**Conclusion:**

Our results suggest that uNAG is a promising biomarker for predicting IgAN remission status.

**Supplementary Information:**

The online version contains supplementary material available at 10.1186/s12882-023-03262-7.

## Background

Immunoglobulin A nephropathy (IgAN) is the most prevalent form of primary glomerulonephritis worldwide [1]. IgAN remains the predominant diagnosis, accounting for 39.5% of glomerular diseases, in Asian descent according to a multiple center survey [2]. The clinical manifestations of IgAN are extremely variable, ranging from persistent asymptomatic microscopic hematuria to rapidly progressive glomerulonephritis [3]. Approximately 15-20% of IgAN patients develop end-stage kidney disease (ESKD) within 10 years and 30-40% within 20 years, making IgAN a leading cause of kidney failure [4]. Considering the substantial risk of adverse outcomes and the significantly heterogeneous clinical course of IgAN, the early identification of patients at high risk of disease progression is of great clinical significance.

Prognostication based on baseline clinical data alone is unreliable. Risk models that use clinical parameters from follow-up studies for at least 2 years may accurately predict risk; however, the time frame required limits the utility of such models in clinical settings [5]. The combination of Oxford MEST scores with clinical data at biopsy has been validated. It allows for early prognostication with an accuracy comparable to that of 2-year clinical follow-up data alone [6]. However, a biopsy is invasive and cannot be performed routinely. As a result, recent research efforts have focused on identifying reliable noninvasive biomarkers to aid in IgAN risk stratification.

N-acetyl-beta-D-glucosaminidase (NAG) is a lysosomal enzyme abundant in proximal tubular epithelial cells (PTEC). It is typically excreted into the urine in very small amounts due to physiological exocytosis [7]. Its relatively high molecular weight (> 130 kDa) precludes glomerular filtration of the enzyme. Thus, elevated urinary NAG (uNAG) excretion is usually attributed to tubular injury with lysosomal membrane damage rather than extrarenal pathologies [8, 9]. Notably, prognosis in IgAN was found to correlate more closely with PTEC and tubulointerstitial injuries than with other histologic lesions [10]. The level of uNAG is reportedly a useful biomarker for various conditions, including diabetic nephropathy, acute kidney injury, primary glomerulonephritis, and exposure to nephrotoxic treatment [11–14]. Few studies have addressed its role in IgAN. Nonetheless, a previous study has demonstrated the ability of uNAG to reflect the severity of glomerulosclerosis and interstitial fibrosis in IgAN [15]. Based on these reports, we hypothesized that uNAG might be a potential biomarker for severity assessment and prognostication in patients with IgAN.

In the present study, we investigated the relationship between uNAG and the clinical and histologic findings, and the remission status of patients with primary IgAN, in parallel with serum cystatin C (sCysC), a widely accepted and highly sensitive indicator of renal function [16]. We incorporated uNAG into the clinical data at biopsy and evaluated their predictive performance in patients with different stages of IgAN. The study aimed to assess whether uNAG is a promising biomarker for the early prediction of IgAN progression.

## Methods

### Study population

**Fig. 1:**
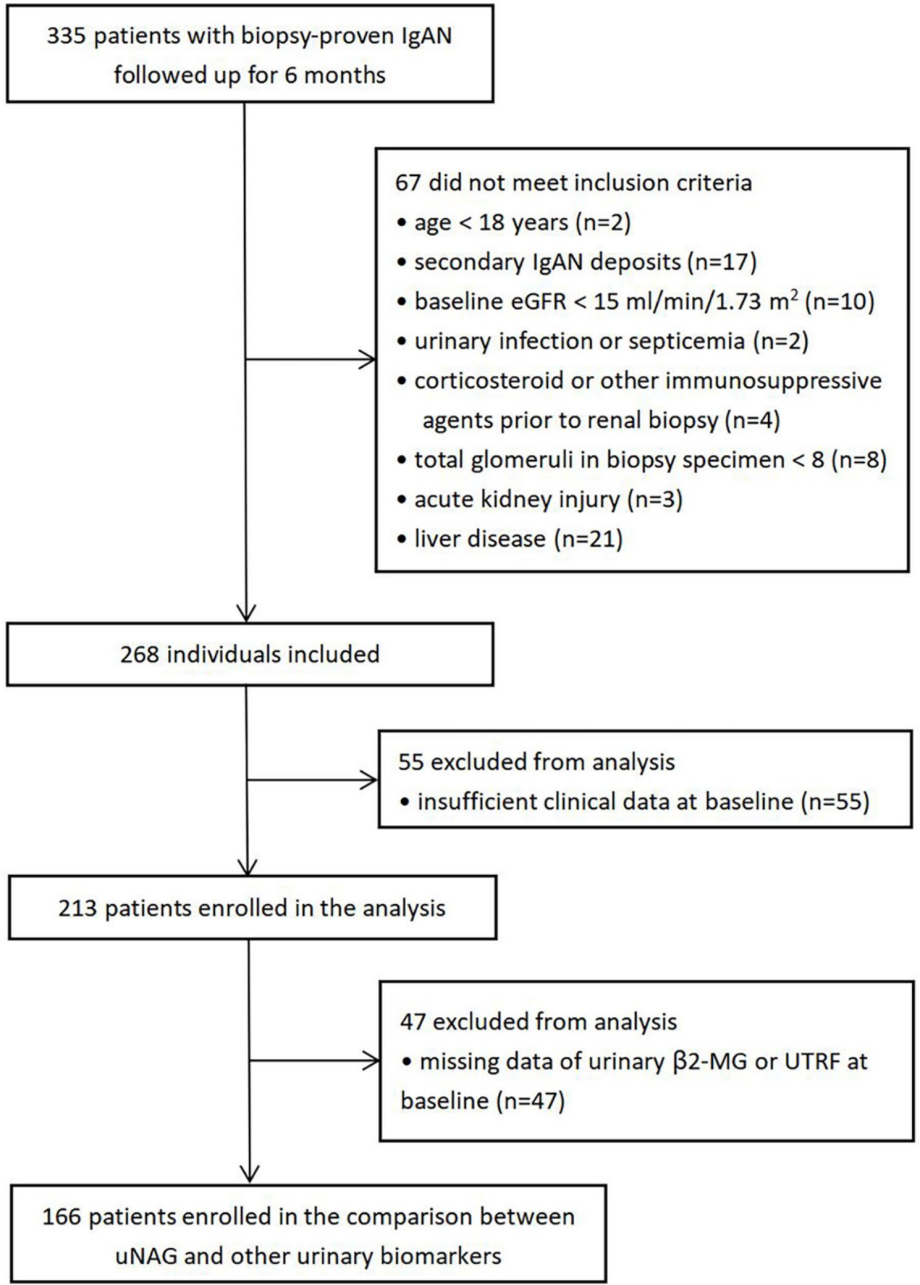
Flow chart for patient selection

We collected data of 335 biopsy-proven IgAN patients, who followed up for at least 6 months, from January 2018 to December 2019 at Zhongshan Hospital, Fudan University. The diagnosis of IgAN was based on light microscopy and immunofluorescence. Patients treated with traditional Chinese medicine before renal biopsy or during pregnancy were excluded at the start of the study. Patients with age under 18 years old (n = 2), eGFR lower than 15 ml/min/1.73 m^2^ on admission (n = 10), or glomeruli count fewer than eight per biopsy section (n = 8) were not admitted. Patients were also excluded when they had a secondary cause of IgA deposition (e.g., systemic lupus erythematosus, rheumatoid arthritis, ankylosing spondylitis, psoriasis) (n = 17), urinary infection or septicemia at present (n = 2), and used steroids or immunosuppressants within 3 months before renal biopsy (n = 4). Patients with acute kidney injury (AKI; n = 3) or liver disease (e.g., hepatitis, cirrhosis, liver tumor) (n = 21) were not enrolled in this study. After excluding those whose baseline clinical data were incomplete (n = 55), 213 patients were finally enrolled in our study (Fig. 1). When comparing the predictive value between uNAG and other urinary biomarkers, 47 patients were excluded because of missing data of urinary β2-microglobulin (β2-MG) or urinary transferrin (UTRF). Written informed consent was obtained from each patient. Procedures were reviewed and approved by the Ethics Committee of Zhongshan Hospital (B2021-027).

### Clinical and histological findings

The following clinical data were collected at the time of renal biopsy: age, sex, body mass index (BMI), mean arterial pressure (MAP), comorbidity (hypertension and diabetes mellitus), blood information, 24-hour proteinuria, urine creatinine and NAG levels. Hypertension was defined as systolic blood pressure (SBP) ≥ 140 mmHg and/or diastolic blood pressure (DBP) ≥ 90 mmHg or using of antihypertensive drugs at the time of renal biopsy. Estimated glomerular filtration rate (eGFR) was calculated using the creatinine-based CKD Epidemiology Collaboration (CKD-EPI) equation. The level of uNAG was measured by rate method using assay kits from Wako. The level of sCysC was measured using enzyme-linked immunosorbent assay (ELISA) kits from Roche. The levels of urinary β2-MG and UTRF were detected by immunity transmission turbidity test using assay kits from Shanghai Kehua Bio-Engineering Co., Ltd. and DiaSys Diagnostic Systems respectively. Urinary levels of biomarkers were all tested in midstream morning urine samples and normalized for urine creatinine.

The histological findings were assessed according to the Oxford classification of IgAN proposed in 2016 [17]. Mesangial hypercellularity (M) was scored as M0 (mesangial score < 0.5) or M1 (mesangial score > 0.5). The scores of endocapillary hypercellularity (E) and segmental sclerosis (S) were classified as 0 (absent) and 1 (present). Tubular atrophy/interstitial fibrosis (T) was scored based on the percentage of area showing this feature and categorized as T0 (≤ 25%), T1 (26-50%) and T2 (> 50%). Crescents (C) were scored as follows: C0 (no crescents), C1 (crescents in at least 1 but < 25% of glomeruli) and C2 (crescents in ≥ 25% of glomeruli). During analysis, T2 and C2 were integrated with T1 and C1 respectively, due to the low amounts of patients with T2 or C2. Global glomerulosclerosis and segmental glomerulosclerosis were presented as the ratio of the number of glomeruli with global or segmental sclerosis to the total number of glomeruli. The severity of tubulointerstitial damage was assessed by a ‘tubulointerstitial score’, which was obtained by adding the scores of tubular atrophy, interstitial fibrosis, and interstitial inflammation, graded by the percentage of cortical area involvement (0, none; 1, 1–25%; 2, 26–50%; 3, > 50%) [18].

### Outcome definitions

Remission status was categorized as complete remission (CR), partial remission (PR) or remission failure (RF), based on the comparison between data recorded at the time of renal biopsy and at the end of six-month follow-up. 24-hour proteinuria and eGFR were used for the evaluation of patients’ outcome. CR was defined as absence of proteinuria (proteinuria ≤ 0.3 g/24 h) and lack of worsening of kidney function (< 30% reduction in eGFR from baseline). PR was defined as proteinuria ≤ 1 g/24 h and lack of worsening of kidney function (< 30% reduction in eGFR from baseline). Proteinuria > 1 g/24 h, reduction ≥ 30% in eGFR over baseline or ESKD indicated RF. eGFR < 15 ml/min per 1.73 m^2^ or requirement for dialysis or transplantation defined ESKD.

### Statistical analyses

IBM SPSS Statistics version 23.0 was used for statistical analysis. Continuous variables were expressed as mean ± standard deviation (SD). Between-group differences were determined using independent sample t test or one-way analysis of variance (ANOVA) when appropriate. Categorical variables were expressed as numbers with percentages in parentheses and compared using the chi-square test. Correlations between parameters were performed by Pearson analysis, while Spearman analysis was used to test the correlation between expression level of biomarkers and the pathological grades. We examined the association between biomarkers and risk stratification of IgAN progression using binary logistic regression analysis in both univariable and multivariable models, in which biomarkers were treated as categorical variables. Covariates included in the model were age, sex, BMI, MAP, 24-hour urine protein, eGFR, MEST-C score and use of renin-angiotensin system inhibition or immunosuppression during follow-up. Adjusted odds ratio (OR) and 95% confidence interval (CI) were obtained for each model. Analyses of receiver operating characteristic curve (ROC) were performed by MedCalc version 20.100. Area Under Curve (AUC) was calculated for quantitative comparison of predictive power between different models. Significant difference between AUC was determined by Delong test. All tests were two-tailed and differences of p value less than 0.05 were considered statistically significant.

## Results

### Baseline characteristics

The detailed clinical and histological data of the 213 patients enrolled are summarized in Table 1. At baseline, mean MAP was 98.77 ± 11.04 mmHg, mean eGFR was 72.85 ± 28.28 mL/min/1.73 m^2^, and mean proteinuria was 1.48 ± 1.21 g/24 h. Patients were categorized into tertiles of uNAG levels (first tertile < 13.29 U/g Cr; second tertile 13.29–22.32 U/g Cr; third tertile > 22.32 U/g Cr). Tables S1 and S2 show baseline characteristics based on sCysC level and remission status, respectively.

**Table 1:** Characteristics of IgAN patients by urinary NAG tertiles at biopsy

Variable^a^	Overall	Urinary NAG (U/g Cr)			*P* ^b^
		T1 (< 13.29)	T2 (13.29–22.32)	T3 (> 22.32)	
No. of patients	213	71	71	71	-
Age, y	41.03 ± 13.33	36.93 ± 10.69	43.89 ± 14.09	42.27 ± 14.08	0.009
Male	118 (55.4%)	43 (60.6%)	43 (60.6%)	32 (45.1%)	0.100
BMI, kg/m^2^	24.41 ± 4.22	24.75 ± 5.69	24.34 ± 3.22	24.14 ± 3.33	0.683
Hypertension	100 (46.9%)	24 (33.8%)	39 (54.9%)	37 (52.1%)	0.023
Diabetes	9 (4.2%)	3 (4.2%)	2 (2.8%)	4 (5.6%)	0.911
MAP, mmHg	98.77 ± 11.04	97.15 ± 10.61	101.05 ± 11.75	98.09 ± 10.49	0.089
Hemoglobin, g/L	128.59 ± 20.02	131.10 ± 18.94	128.86 ± 20.90	125.82 ± 20.09	0.289
Serum albumin, g/L	39.00 ± 4.68	40.23 ± 4.56	39.41 ± 3.84	37.37 ± 5.14	0.001
CRP, mg/dL	1.65 ± 2.79	1.22 ± 1.65	1.97 ± 3.86	1.78 ± 2.39	0.504
Serum creatinine, mg/dL	1.13 ± 0.53	0.97 ± 0.34	1.19 ± 0.51	1.23 ± 0.66	0.017
eGFR, mL/min/1.73m^2^	72.85 ± 28.28	84.15 ± 25.10	68.06 ± 28.12	66.34 ± 28.38	< 0.001
Proteinuria, g/24 h	1.48 ± 1.21	1.04 ± 1.02	1.33 ± 0.87	2.07 ± 1.45	< 0.001
Use ACEI/ARBs at biopsy	176 (82.6%)	57 (80.3%)	61 (85.9%)	58(81.7%)	0.654
Oxford MEST-C					
M1	186 (87.3%)	56 (78.9%)	65 (91.5%)	65 (91.5%)	0.032
E1	32 (15.0%)	8 (11.3%)	11 (15.5%)	13 (18.3%)	0.497
S1	116 (54.5%)	29 (40.8%)	39 (54.9%)	48 (67.6%)	0.006
T1-2	101 (47.4%)	24 (33.8%)	37 (52.1%)	40 (56.3%)	0.017
C1-2	84 (39.4%)	24 (33.8%)	31 (43.7%)	29 (40.8%)	0.465

Abbreviations: BMI, body mass index; MAP, mean arterial blood pressure; CRP, C-reactive protein; eGFR, estimated glomerular filtration rate; ACEI, Angiotensin-converting enzyme inhibitors; ARBs, Angiotensin II receptor blockers; MEST-C, histologic score based on mesangial hypercellularity, the presence of endocapillary proliferation, segmental glomerulosclerosis/adhesion, and severity of tubular atrophy/interstitial fibrosis, and crescents formation; T, tertile

^a^Continuous variables are expressed as mean ± standard deviation. Categorical variables are expressed as number (percent)

^b^Comparing the covariated across the 3 urinary NAG categories

### Correlation of uNAG and sCysC levels with clinical parameters

We first explored the relationship between uNAG levels and several clinical features. The results showed that uNAG levels were significantly positively correlated with serum creatinine (r = 0.150, p = 0.029), 24-hour proteinuria (r = 0.346, p < 0.001), serum cholesterol (r = 0.174, p = 0.011) and serum triglyceride (r = 0.241, p < 0.001), but negatively correlated with eGFR (r = -0.196, p = 0.004) and serum albumin at baseline (r = -0.307, p < 0.001; Table 2). No relationship was found between uNAG and MAP, hemoglobin, and other lipid indices tested. Table S3 listed the results of the correlation analysis for sCysC, of which most were significant, suggesting the clinical value of sCysC in IgAN patients. These results indicated that increased uNAG levels are associated with more severe clinical manifestations.

**Table 2:** Correlations between expression levels of urinary NAG and clinical parameters at baseline

Clinical parameters	Urinary NAG (U/g Cr)		
	Correlation	r	P value
Serum creatinine, mg/dl	Pos	0.150	0.029
eGFR, mL/min/1.73m^2^	Neg	-0.196	0.004
Proteinuria, g/24 hour	Pos	0.346	< 0.001
MAP, mmHg	No Sig	0.067	0.327
Hemoglobin, g/L	No Sig	-0.047	0.493
Serum albumin, g/L	Neg	-0.307	< 0.001
Serum cholesterol, g/L	Pos	0.174	0.011
Serum triglyceride, g/L	Pos	0.241	<0.001
LDL-cholesterol, g/L	No Sig	0.050	0.465
HDL-cholesterol, g/L	No Sig	0.015	0.829
Serum IgA, g/L	No Sig	0.037	0.592
Serum C3, g/L	No Sig	-0.025	0.712
Serum uric acid, µmol/L	No Sig	0.021	0.760
Serum erythropoietin, IU/L	No Sig	-0.113	0.099

Abbreviations: NAG, N-acetyl-beta-D-glucosaminidase; r, correlation coefficient; Pos, positive correlation; Neg, negative correlation; No Sig, no significant correlation; eGFR, estimated glomerular filtration rate; MAP, mean arterial blood pressure

### Relationship of uNAG and sCysC levels with histological severity

**Fig. 2:**
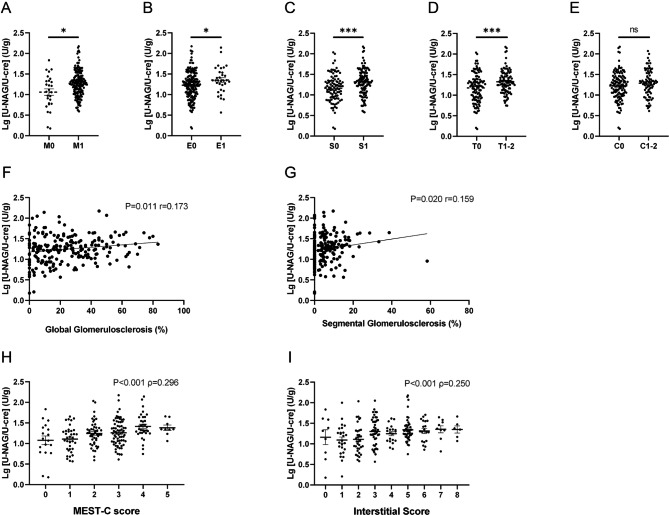
Relationship between urinary N-acetyl-beta-D-glucosaminidase (uNAG) level and patient histology. (**A**-**E**) Association between uNAG level and Oxford classification. *P < 0.05, ***P < 0.001. (**F**) Correlation between uNAG level and global glomerulosclerosis. (**G**) Correlation between uNAG level and segmental glomerulosclerosis. (**H**) Correlation between uNAG level and MEST-C score. (**I**) Correlation between uNAG level and interstitial score; each dot represents a value from an individual patient. Coefficients of correlation (r for Pearson analysis and ρ for Spearman analysis, respectively) and p values are shown

We first analyzed the association between uNAG levels and the Oxford classification. Patients with mesangial hypercellularity (p = 0.015; Fig. 2A), endocapillary hypercellularity (p = 0.046; Fig. 2B), segmental sclerosis (p = 0.001; Fig. 2C), and relatively severe tubulointerstitial damage (T1-2, p = 0.001; Fig. 2D) showed markedly higher levels of uNAG. The level of uNAG did not significantly differ between patients scored C0 and C1-2 (Fig. 2E). We found that uNAG levels were correlated with global glomerulosclerosis score (r = 0.173, p = 0.011; Fig. 2F) and segmental glomerulosclerosis score (r = 0.159, p = 0.020; Fig. 2G). Furthermore, significant relationships between uNAG levels and both MEST-C scores (ρ = 0.296, p < 0.001; Fig. 2H) and interstitial scores (ρ = 0.250, p < 0.001; Fig. 2I) were revealed using Spearman analysis.

We also assessed the relationship between sCysC levels and the Oxford classification. The levels of sCysC increased in patients scored M1 (p = 0.002; Supplementary Fig. 1A) and T1-2 (p < 0.0001; Supplementary Fig. 1D), but not in patients scored E1 or S1. The levels of sCysC were also correlated with global glomerulosclerosis score (r = 0.603, p < 0.001; Supplementary Fig. 1F), MEST-C score (ρ = 0.363, p < 0.001; Supplementary Fig. 1H) and interstitial score (ρ = 0.690, p < 0.001; Supplementary Fig. 1I), but not with segmental glomerulosclerosis score. Taken together, uNAG and sCysC levels are able to reflect the histological states of IgAN. Supplementary Fig. 2 showed representative images of kidney tissue sections of IgAN patients evaluated according to the Oxford classification.

### Association of biomarkers with IgAN progression

We first analyzed the relationship between the levels of uNAG and sCysC and the risk of IgAN progression. In univariable models, uNAG and sCysC showed significant associations with IgAN progression. However, the association of sCysC levels was no longer significant after adjusting for baseline clinical parameters (age, sex, BMI, MAP, 24-hour urine protein, and eGFR) and MEST-C score in Model 2. In contrast, uNAG outperformed sCysC in each model and was still strongly associated with a higher risk of IgAN progression after further adjusting for using renin-angiotensin system inhibition or immunosuppression during follow-up in Model 3, showing a 4.32-fold greater risk of IgAN progression in patients with uNAG levels > 22.32 U/g Cr over those whose biomarker did not reach this threshold (Table 2). We also examined the relationship in patients of different subgroups determined by baseline eGFR data. The ability of uNAG to predict progression remained in patients with eGFR ≥ 60 ml/min/1.73 m^2^ (OR = 4.01, p = 0.020). However, for patients with baseline eGFR < 60 ml/min/1.73 m^2^, uNAG was no longer independently associated with remission status in Model 2, suggesting that uNAG may be more useful for patients with a relatively early stage of IgAN (Table 3). Table S4 presented the multivariable logistic analyses for predicting risk of IgAN progression of urinary β2-MG, a typical marker of tubulointerstitial malfunction, and UTRF, which is considered as glomerular injury marker.

**Table 3:** Multivariable logistic analyses of urinary NAG and serum CysC for predicting risk of IgAN progression

	Cut Points	Remission failure %	Unadjusted OR (95% Cl); *P*	Adjusted OR (95% Cl)		
				Model 1^a^, *P*	Model 2^b^, *P*	Model 3^c^, *P*
Urinary NAG (U/g Cr)						
T1 + T2 (n = 142)	≤ 22.32	9.2	1.0 (referent)	1.0 (referent)	1.0 (referent)	1.0 (referent)
T3 (n = 71)	> 22.32	42.3	7.26 (3.47–15.21); <0.001	7.18 (3.39–15.22); <0.001	4.41 (1.94–9.99); <0.001	4.32 (1.87–9.96); 0.001
Serum CysC (mg/L)						
T1 + T2 (n = 142)	≤ 1.34	13.4	1.0 (referent)	1.0 (referent)	1.0 (referent)	1.0 (referent)
T3 (n = 71)	> 1.34	33.8	3.31 (1.66–6.59); 0.001	4.33 (2.00-9.36); < 0.001	1.42 (0.43–4.69); 0.561	1.32 (0.39–4.46); 0.655

Abbreviations: Cl, confidence interval; Cr, creatinine; OR, Odds ratio; BMI, body mass index; eGFR, estimated glomerular filtration rate; MAP, mean arterial blood pressure; MEST-C, histologic score based on mesangial hypercellularity, the presence of endocapillary proliferation, segmental glomerulosclerosis/adhesion, and severity of tubular atrophy/interstitial fibrosis, and crescents formation; T, tertile

^a^Model 1 adjusted for age, sex, MAP, BMI

^b^Model 2 adjusted for covariates in model 1 plus 24-hour proteinuria, eGFR and Oxford MEST-C score

^c^Model 3 adjusted for covariates in model 1 and 2 plus use of renin-angiotensin system inhibition and immunosuppression during follow-up

**Table 4:** Multivariable logistic analyses of urinary NAG for predicting risk of IgAN progression in subgroups of eGFR

	Cut Points	Remission failure %	Unadjusted OR (95% Cl); *P*	Adjusted OR (95% Cl)		
				Model 1^a^, *P*	Model 2^b^, *P*	Model 3^c^, *P*
Subgroup with eGFR ≥ 60 ml/min/1.73 m^2^ (n = 142)						
T1 + T2 urinary NAG (n = 101)	≤ 22.32	6.9	1.0 (referent)	1.0 (referent)	1.0 (referent)	1.0 (referent)
T3 urinary NAG (n = 41)	> 22.32	31.7	6.23 (2.27–17.14); <0.001	5.62 (1.97-16.00); 0.001	5.05 (1.66–15.37); 0.004	4.01 (1.24–12.93); 0.020
Subgroup with eGFR < 60 ml/min/1.73 m^2^ (n = 71)						
T1 + T2 urinary NAG (n = 41)	≤ 22.32	14.6	1.0 (referent)	1.0 (referent)	1.0 (referent)	1.0 (referent)
T3 urinary NAG (n = 30)	> 22.32	56.7	7.63 (2.47–23.56); <0.001	7.85 (2.38–25.87); 0.001	3.16 (0.44–22.43); 0.250	3.38 (0.46–25.01); 0.233

Abbreviations: Cl, confidence interval; Cr, creatinine; OR, Odds ratio; BMI, body mass index; eGFR, estimated glomerular filtration rate; MAP, mean arterial blood pressure; MEST-C, histologic score based on mesangial hypercellularity, the presence of endocapillary proliferation, segmental glomerulosclerosis/adhesion, and severity of tubular atrophy/interstitial fibrosis, and crescents formation; T, tertile

^a^Model 1 adjusted for age, sex, MAP, BMI

^b^Model 2 adjusted for covariates in model 1 plus 24-hour proteinuria, eGFR and Oxford MEST-C score

^c^Model 3 adjusted for covariates in model 1 and 2 plus use of renin-angiotensin system inhibition and immunosuppression during follow-up

### Use of uNAG with clinical and histological data for predicting risk of IgAN progression

**Fig. 3:**
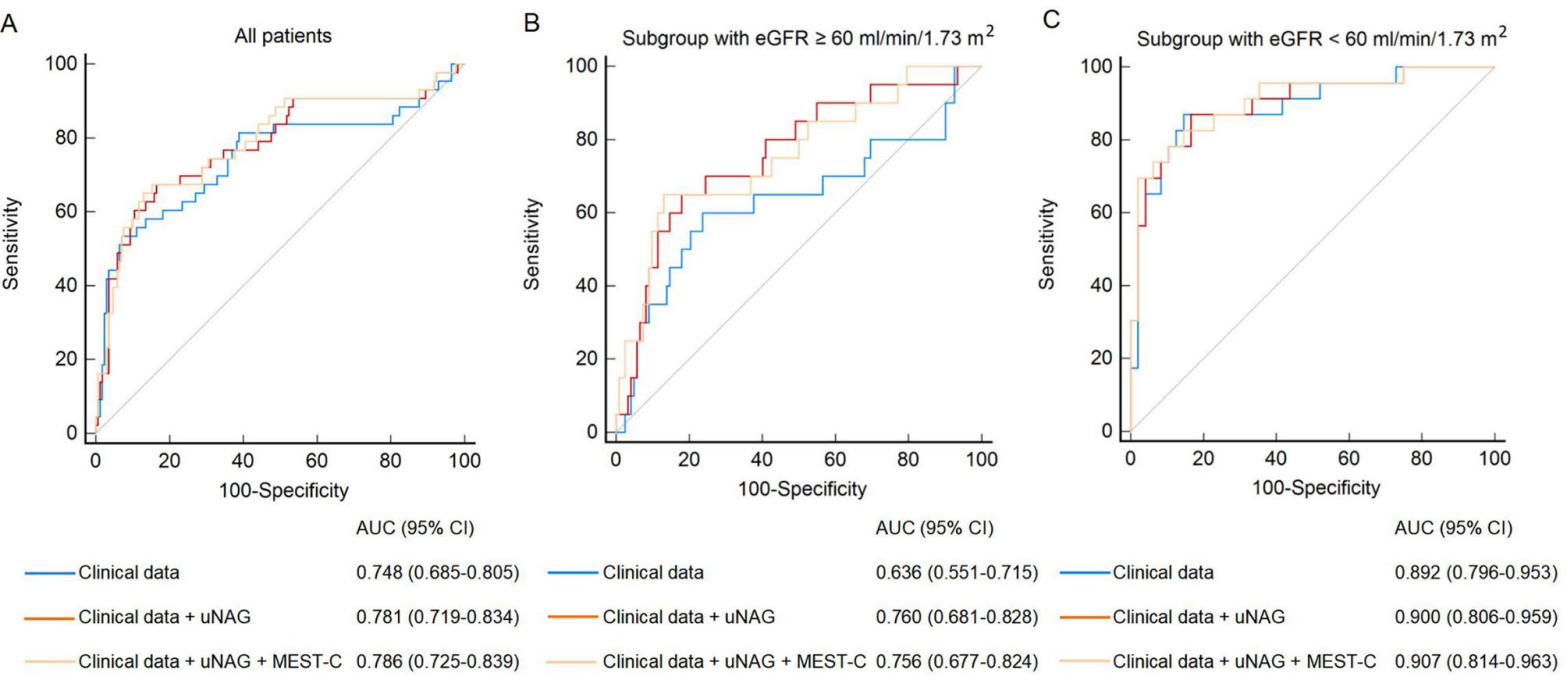
ROC analysis for models predicting IgAN remission status. (**A**) In the whole cohort (n = 213). (**B**) In patients with baseline eGFR ≥ 60 ml/min/1.73 m^2^ (n = 142). (**C**) In patients with baseline eGFR < 60 ml/min/1.73 m^2^ (n = 71). The blue line represents the model based on clinical data at biopsy alone; the red line represents the model based on the combination of clinical data and uNAG level at biopsy; the yellow line represents the model using baseline clinical data, uNAG level and MEST-C score. Clinical data were MAP, 24-hour proteinuria and eGFR

ROC analysis was used to evaluate the performance of uNAG and sCysC in predicting IgAN remission status. When applied to the whole patient cohort, uNAG (AUC 0.771, 95% CI, 0.709–0.826) showed much stronger predictive power than sCysC (AUC 0.677, 95% CI, 0.610–0.739). We also tested whether combining uNAG levels with clinical parameters at biopsy (MAP, 24-hour proteinuria, and eGFR) improved predictive ability over clinical data alone. As shown in Fig. 3A, adding uNAG levels to the model composed of three clinical indicators (AUC 0.781, 95% CI, 0.719–0.834) improved its predictive performance (AUC 0.748, 95% CI, 0.685–0.805). However, adding the MEST-C score to the model containing uNAG levels and clinical characteristics (AUC 0.786, 95% CI, 0.725–0.839) did not significantly improve risk stratification (DeLong test, p = 0.42). ROC analysis was also performed for urinary β2-MG (AUC 0.699, 95% CI, 0.623–0.767) and UTRF (AUC 0.799, 95% CI, 0.730–0.857) in the 166 patients with available data.

When applied to the subgroup with eGFR ≥ 60 ml/min/1.73 m^2^, the combination of uNAG level and clinical data resulted in an AUC of 0.760 (95% CI, 0.681–0.828), demonstrating strongly better predictive performance than clinical data alone (AUC 0.636, 95% CI, 0.551–0.715; DeLong test, p = 0.05). It was also more accurate than the model further integrated with MEST-C score (AUC 0.756, 95% CI, 0.677–0.824; Fig. 3B). In the subgroup with baseline eGFR < 60 ml/min/1.73 m^2^, combination of uNAG level with clinical data showed higher AUC (AUC 0.900, 95% CI, 0.806–0.959) than those of whole cohort and subgroup with eGFR ≥ 60 ml/min/1.73 m^2^, however, addition of uNAG level did not exhibit satisfactory predictive value compared with mere clinical data (AUC 0.892, 95% CI, 0.796–0.953) or the model further integrated with MEST-C score (AUC 0.907, 95% CI, 0.814–0.963) in this subgroup (Fig. 3C).

## Discussion

The identification of biomarkers for IgAN progression generally considers the multi-hit pathogenesis of IgAN or the degree of kidney damage [19]. However, these indicators are not routinely examined. Elevated uNAG levels usually indicate renal tubulointerstitial injury. In the present study, we evaluated the predictive value of uNAG in patients with IgAN. We also compared the predictive effect with sCysC, a key indicator of renal damage [12]. We demonstrated that uNAG level measured at biopsy is a promising predictor of IgAN progression. To our knowledge, this is the first study to assess the prognostic value of uNAG in IgAN patients.

The present study found that baseline uNAG levels were significantly correlated with several traditional risk factors of IgAN progression, similar as the well-recognized marker sCysC. However, in both univariate and multivariate models, uNAG levels delivered better predictive performance than sCysC levels.

Furthermore, we found that uNAG exhibited a much stronger predictive power in patients with early stage of IgAN (baseline eGFR ≥ 60 ml/min/1.73 m^2^). ROC analysis revealed that adding uNAG to clinical data at biopsy resulted in the greatest improvement in risk prediction. However, in patients with eGFR < 60 ml/min/1.73 m^2^, the predictive power of uNAG disappeared. These results may be attributed to uNAG mainly originating from damaged PTECs and rapidly increasing approximately 12 h after tubular injury [9, 12]. In IgAN, PTECs were confirmed to be strongly affected at an early stage, making uNAG capable of providing prognostic information during this period [20]. However, chronic kidney pathologies are characterized by tubular atrophy and interstitial fibrosis [21]. During this period many tubules are already replaced by matrix proteins, so uNAG cannot serve as a predictor of IgAN progression at a relatively later stage. Other tubular injury markers had similar limitations. A study of 2,466 general CKD patients with eGFR ranging from 20 to 70 ml/min/1.73 m^2^ proved adding renal tubular injury markers to the clinical model including serum creatinine-based eGFR and urinary albumin/creatinine ratio did not improve risk prediction, in which kidney injury molecule-1 (KIM-1), neutrophil gelatinase-associated lipocalin (NGAL), liver fatty acid binding protein (L-FABP) and NAG were examined [22]. Thus, urinary markers associated with tubular cell damage may be more useful for populations with preserved renal function.

An interesting finding of our study was that adding MEST-C score to the model containing baseline uNAG and clinical data did not improve predictive ability in the whole cohort. Conversely, it led to a decline in its predictive performance for patients with eGFR ≥ 60 ml/min/1.73 m^2^ suggesting the possibility to develop a risk prediction tool with sufficient accuracy using noninvasive biomarkers. Although risk stratification based on clinical and histological data has been well established and externally validated, it necessitates histological scores obtained concurrently with clinical data [23, 24]. However, the application of biopsy is often limited for several reasons, including different regional biopsy policies, lack of necessary medical resources in remote hospitals, high risk of post-biopsy complications [1, 2, 18]. Owing to inconclusive histological findings, it is still difficult to predict the risk of disease progression even after biopsy [25]. Accordingly, there is an urgent need to develop noninvasive methods in supplement with renal biopsy to assess risk stratification and guide treatment. Our results demonstrated that the combination of uNAG levels and clinical data could develop a strong and noninvasive prediction tool without pathological data in IgAN patients with relatively preserved renal function. Urine samples are easy to obtain and measurement of uNAG is clinically available worldwide. Meanwhile, urine NAG remains relatively stable with minimal diurnal variations [26]. Thus, uNAG might be an excellent biomarker for the risk prediction of IgAN progression.

Our study has several limitations. First, our patients were from a single center, and the number of subjects (n = 213) may limit the generalizability of our findings; therefore, our results require validation in larger and independent IgAN cohorts. Second, the follow-up time was inadequate. As such, we could not investigate the association between uNAG and long-term renal outcomes. In this study we combined 24-hour proteinuria at the end of six-month follow-up, reduction of eGFR and occurence of ESKD to determine remission status. 24-hour proteinuria is a traditional adverse prognostic factor in IgAN. A study revealed that proteinuria during follow-up was the most important predictor of the rate of GFR decline and remission of proteinuria improved prognosis in IgAN [27]. Both 24-hour proteinuria and decreased eGFR were associated with a higher risk of kidney function decline in IgAN [28]. Combination of eGFR decline and ESKD were commonly used to define progression in IgAN [29–31]. Taken together, we believe that the short-term renal outcome we defined could partially reflect long-term outcome. Third, we did not have complete follow-up data to examine the relationship between uNAG and clinical and histological findings over time. Thus, it remains speculative whether uNAG is useful for evaluating disease severity in patients with IgAN. Finally, besides of uNAG, there are several tubular markers, while elevation of uNAG levels is common in active renal diseases with tubulointerstitial involvement, including AKI, diabetic nephropathy, and various primary glomerulonephritis [11–13]. UNAG levels show promise as a biomarker in IgAN, although it is non-specific. Further studies would be necessary to validate our data. 

## Conclusion

In conclusion, our study revealed that uNAG level was an independent and strong predictor of IgAN remission status. Adding uNAG levels to baseline clinical data produced a promising prediction model of disease progression among IgAN patients at a relatively early stage.

## Electronic supplementary material

Below is the link to the electronic supplementary material.

Supplementary Material 1

Supplementary Material 2

## Data Availability

The data underlying this article will be shared on reasonable request to the corresponding author.

## Abbreviations

ANOVA: Analysis of variance
AUC: Area Under Curve
BMI: Body mass index
CI: Confidence interval
CKD-EPI: Chronic Kidney Disease Epidemiology Collaboration
CR: Complete remission
DBP: Diastolic blood pressure
eGFR: Estimated glomerular filtration rate
ELISA: Enzyme-linked immunosorbent assay
ESKD: End-stage kidney disease
IgAN: Immunoglobulin A nephropathy
KIM-1: Kidney injury molecule-1
L-FABP: Liver fatty acid binding protein
MAP: Mean arterial pressure
NAG: N-acetyl-beta-D-glucosaminidase
NGAL: Neutrophil gelatinase-associated lipocalin
OR: Odds ratio
PR: Partial remission
PTEC: Proximal tubular epithelial cells
RF: Remission failure
ROC: Receiver operating characteristic curve
SBP: Systolic blood pressure
sCysC: Serum cystatin C
SD: Standard deviation
uNAG: Urinary NAG
UTRF: Urinary transferrin
β2-MG: β2-microglobulin
