# Supplementary material for: Urinary N-Acetyl-Beta-D-Glucosaminidase levels predict immunoglobulin a nephropathy remission status
Source: BMC Nephrol. 2023 Jul 14;24:208. doi: 10.1186/s12882-023-03262-7 (PMC10347709; doi:10.1186/s12882-023-03262-7)

**Supplementary Figure 1**. Relationship between serum Cystatin C (sCysC) level and patient histology. **(A-E)** Association between sCysC level and Oxford classification. **P < 0.01, ****P < 0.0001. **(F)** Correlation between sCysC level and global glomerulosclerosis. **(G)** Correlation between sCysC level and segmental glomerulosclerosis. **(H)** Correlation between sCysC level and MEST-C score. **(I)** Correlation between sCysC level and interstitial score; each dot represents a value from an individual patient. Coefficients of correlation (r for Pearson analysis and ρ for Spearman analysis, respectively) and p values are shown.


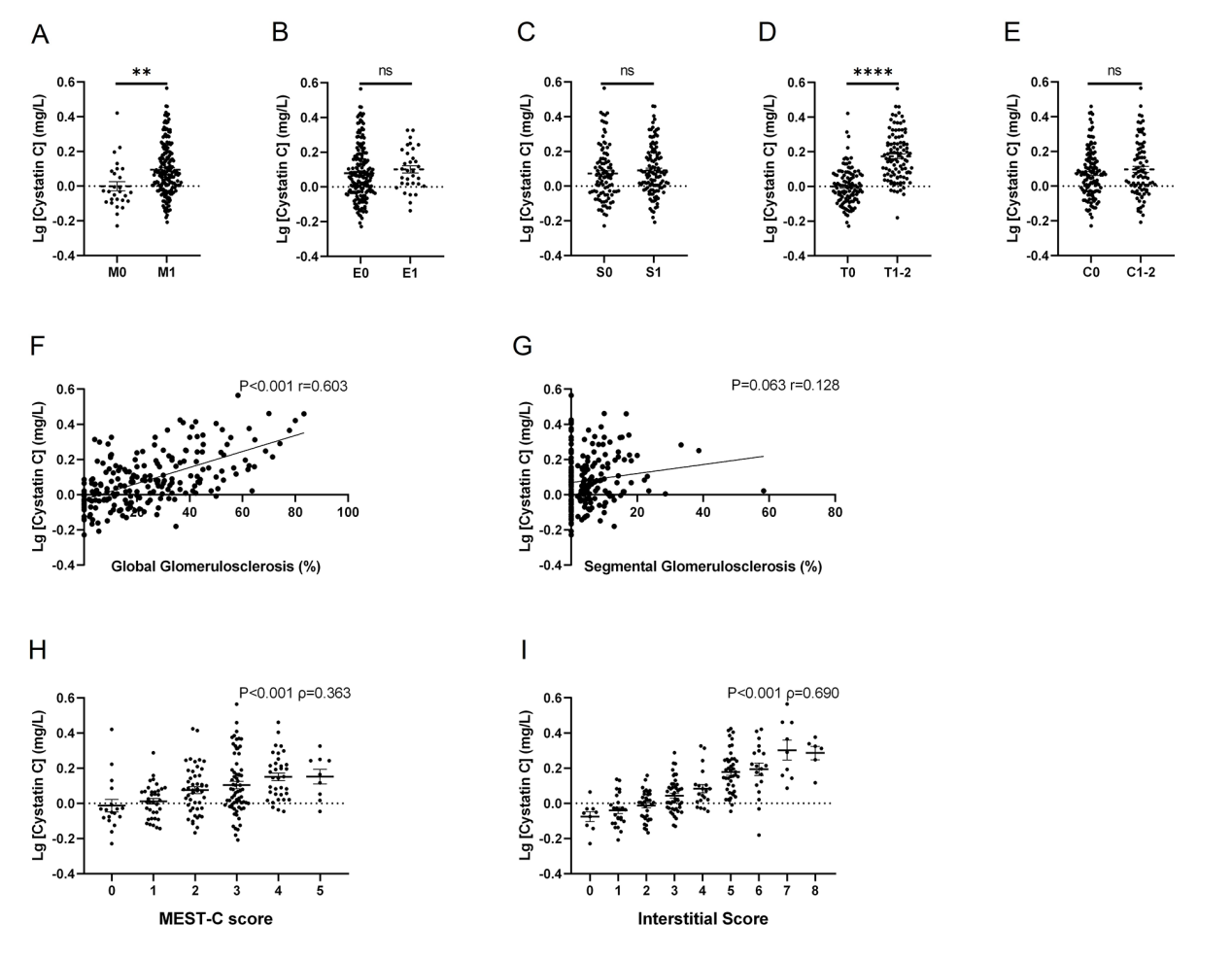


**Supplementary Figure 2**. Pathology pictures of kidney biopsy sections of IgAN patients evaluated according to the Oxford classification. Representative images of kidney tissue sections stained with periodic acid-Schiff (PAS) of IgAN patients scored as **(A)** M1, **(B)** E1, **(C)** S1 and **(D)** C1. Bar: 50 µm.


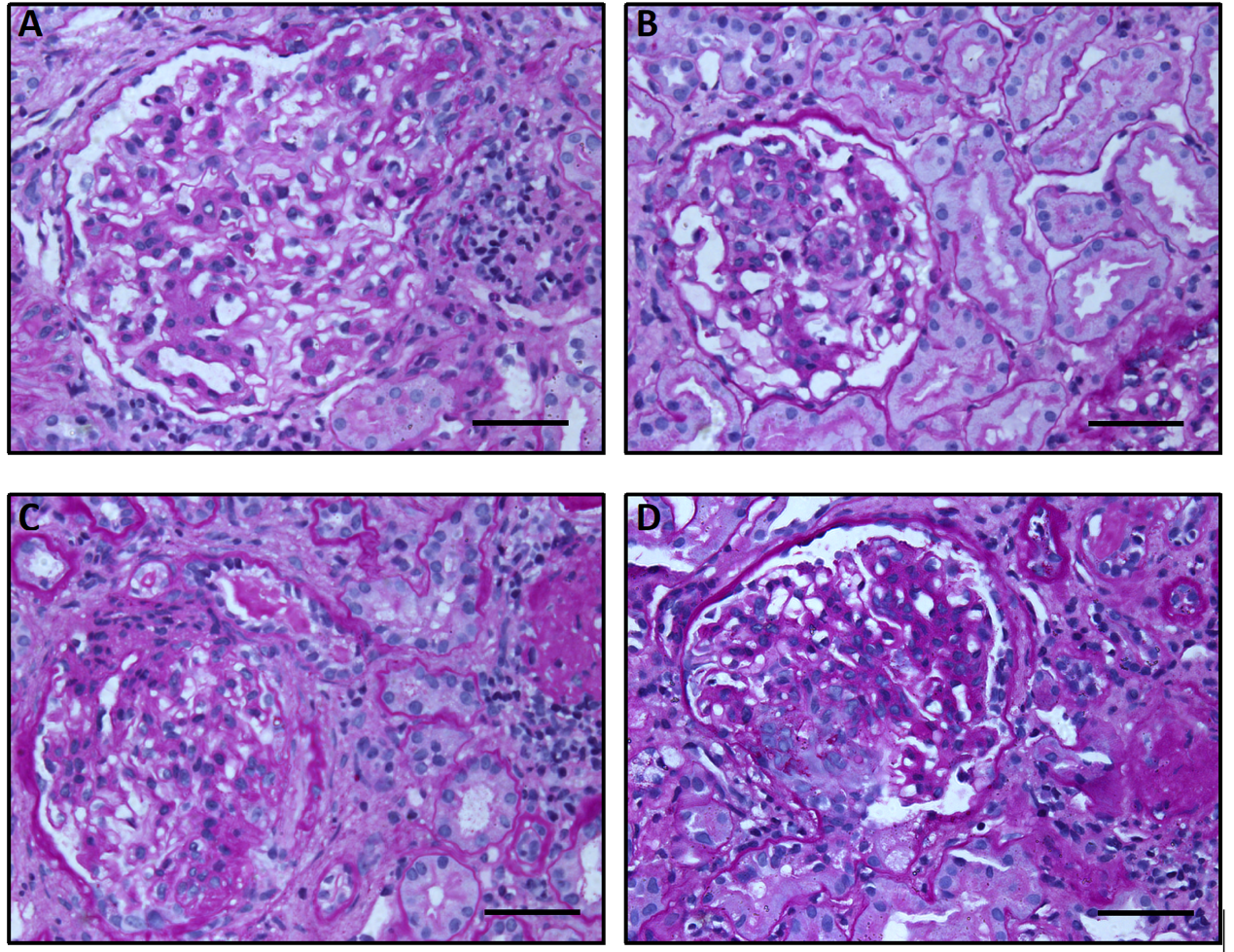

Supplement: Supplementary file 1 — Supplementary Material 1 [file 12882_2023_3262_MOESM1_ESM.docx]
