# Supplementary material for: Urinary N-Acetyl-Beta-D-Glucosaminidase levels predict immunoglobulin a nephropathy remission status
Source: BMC Nephrol. 2023 Jul 14;24:208. doi: 10.1186/s12882-023-03262-7 (PMC10347709; doi:10.1186/s12882-023-03262-7)
Supplement: Supplementary file 2 — Supplementary Material 2 [file 12882_2023_3262_MOESM2_ESM.docx]

**Supplementary Table 1. Characteristics of IgAN patients by serum CysC tertiles at biopsy**

|  |  | | **Serum CysC (mg/L)** | |  |
| --- | --- | --- | --- | --- | --- |
| **Variable^a^** | **Overall** | **T1 (<1.01)** | **T2 (1.01-1.34)** | **T3 (>1.34)** | ***P* ^b^** |
| No. of patients | 213 | 71 | 71 | 71 | - |
| Age, y | 41.03±13.33 | 35.92±10.67 | 40.35±11.86 | 46.82±14.92 | <0.001 |
| Male | 118 (55.4%) | 30 (42.3%) | 40 (56.3%) | 48 (67.6%) | 0.010 |
| BMI, kg/m^2^ | 24.41±4.22 | 24.44±5.65 | 24.03±3.28 | 24.78±3.34 | 0.580 |
| Hypertension | 100 (46.9%) | 14 (19.7%) | 34 (47.9%) | 52 (73.2%) | <0.001 |
| Diabetes | 9 (4.2%) | 1 (1.4%) | 5 (7.0%) | 3 (4.2%) | 0.305 |
| MAP, mmHg | 98.77±11.04 | 95.64±9.95 | 99.29±11.15 | 101.37±11.35 | 0.007 |
| Hemoglobin, g/L | 128.59±20.02 | 128.65±17.67 | 133.66±20.06 | 123.46±21.13 | 0.009 |
| Serum albumin, g/L | 39.00±4.68 | 39.62±5.53 | 39.62±3.73 | 37.76±4.41 | 0.023 |
| CRP, mg/dL | 1.65±2.79 | 1.24±1.65 | 1.20±1.59 | 2.53±4.15 | 0.045 |
| Serum creatinine, mg/dL | 1.13±0.53 | 0.74±0.16 | 1.01±0.19 | 1.64±0.59 | <0.001 |
| eGFR, mL/min/1.73m^2^ | 72.85±28.28 | 102.14±17.13 | 72.79±13.51 | 43.62±14.47 | <0.001 |
| Proteinuria, g/24h | 1.48±1.21 | 1.32±1.38 | 1.21±0.92 | 1.92±1.19 | 0.001 |
| Use ACEI/ARBs at biopsy | 176 (82.6%) | 61 (85.9%) | 62 (87.3%) | 53(74.6%) | 0.092 |
| Oxford MEST-C |  |  |  |  |  |
| M1 | 186 (87.3%) | 53 (74.6%) | 66 (93.0%) | 67 (94.4%) | <0.001 |
| E1 | 32 (15.0%) | 6 (8.5%) | 13 (18.3%) | 13 (18.3%) | 0.165 |
| S1 | 116 (54.5%) | 34 (47.9%) | 42 (59.2%) | 40 (56.3%) | 0.374 |
| T1-2 | 101 (47.4%) | 7 (9.9%) | 35 (49.3%) | 59 (83.1%) | <0.001 |
| C1-2 | 84 (39.4%) | 28 (39.4%) | 24 (33.8%) | 32 (45.1%) | 0.389 |
| Abbreviations: BMI, body mass index; MAP, mean arterial blood pressure; CRP, C-reactive protein; eGFR, estimated glomerular filtration rate; ACEI, Angiotensin-converting enzyme inhibitors; ARBs, Angiotensin II receptor blockers; MEST-C, histologic score based on mesangial hypercellularity, the presence of endocapillary proliferation, segmental glomerulosclerosis/adhesion, and severity of tubular atrophy/interstitial fibrosis, and crescents formation; T, tertile.  ^a^Continuous variables are expressed as mean ± standard deviation. Categorical variables are expressed as number (percent).  ^b^Comparing the covariated across the 3 serum CysC categories. | | | | | |

**Supplementary Table 2. Characteristics of IgAN patients by remission status**

| **Variable^a^** | **Overall** | **CR** | **PR** | **RF** | ***P* ^b^** |
| --- | --- | --- | --- | --- | --- |
| No. of patients | 213 | 70 | 100 | 43 | - |
| Age, y | 41.03±13.33 | 39.34±11.52 | 41.83±14.34 | 41.91±13.66 | 0.742 |
| Male | 118 (55.4%) | 40 (57.1%) | 58 (58.0%) | 20 (46.5%) | 0.420 |
| BMI, kg/m^2^ | 24.41±4.22 | 24.36±5.60 | 24.40±3.15 | 24.54±3.90 | 0.974 |
| Hypertension | 100 (46.9%) | 28 (40.0%) | 47 (47.0%) | 25 (58.1%) | 0.172 |
| Diabetes | 9 (4.2%) | 3 (4.3%) | 4 (4.0%) | 2 (4.7%) | 1.000 |
| MAP, mmHg | 98.77±11.04 | 95.95±12.05 | 100.78±10.57 | 98.67±9.51 | 0.019 |
| Hemoglobin, g/L | 128.59±20.02 | 128.83±19.58 | 130.52±21.42 | 123.72±16.69 | 0.176 |
| Serum albumin, g/L | 39.00±4.68 | 39.60±5.45 | 39.40±3.69 | 37.09±5.00 | 0.010 |
| CRP, mg/dL | 1.65±2.79 | 1.50±2.65 | 1.77±3.08 | 1.64±2.33 | 0.818 |
| Serum creatinine, mg/dL | 1.13±0.53 | 0.98±0.34 | 1.10±0.38 | 1.46±0.85 | 0.008 |
| eGFR, mL/min/1.73m^2^ | 72.85±28.28 | 81.97±26.21 | 72.11±25.56 | 59.72±32.43 | <0.001 |
| Proteinuria, g/24h | 1.48±1.21 | 1.10±1.38 | 1.40±0.86 | 2.30±1.27 | <0.001 |
| Use ACEI/ARBs at biopsy | 176 (82.6%) | 58 (82.9%) | 86 (86.0%) | 32 (74.4%) | 0.245 |
| Urinary NAG, U/g Cr | 23.68±21.74 | 19.69±20.57 | 20.40±16.83 | 37.80±27.70 | <0.001 |
| Serum CysC, mg/L | 1.29±0.50 | 1.13±0.35 | 1.26±0.42 | 1.61±0.70 | <0.001 |
| Oxford MEST-C |  |  |  |  |  |
| M1 | 186 (87.3%) | 56 (80.0%) | 90 (90.0%) | 40 (93.0%) | 0.071 |
| E1 | 32 (15.0%) | 11 (15.7%) | 10 (10.0%) | 11 (25.6%) | 0.056 |
| S1 | 116 (54.5%) | 32 (45.7%) | 54 (54.0%) | 30 (69.8%) | 0.044 |
| T1-2 | 101 (47.4%) | 26 (37.1%) | 46 (46.0%) | 29 (67.4%) | 0.007 |
| C1-2 | 84 (39.4%) | 23 (32.9%) | 43 (43.0%) | 18 (41.9%) | 0.386 |
| Abbreviations: CR, complete remission; PR, partial remission; RF, remission failure; BMI, body mass index; MAP, mean arterial blood pressure; CRP, C-reactive protein; eGFR, estimated glomerular filtration rate; ACEI, Angiotensin-converting enzyme inhibitors; ARBs, Angiotensin II receptor blockers; MEST-C, histologic score based on mesangial hypercellularity, the presence of endocapillary proliferation, segmental glomerulosclerosis/adhesion, and severity of tubular atrophy/interstitial fibrosis, and crescents formation.  ^a^Continuous variables are expressed as mean ± standard deviation. Categorical variables are expressed as number (percent).  ^b^Comparing the covariated across 3 remission status (CR, PR and RF). | | | | | |

**Supplementary Table 3. Correlations between expression levels of serum CysC and clinical parameters at baseline**

| **Clinical**  **parameters** | **Serum CysC (mg/L)** | | |
| --- | --- | --- | --- |
|  | **Correlation** | **r** | **P value** |
| Serum creatinine, mg/dl | Pos | 0.884 | <0.001 |
| eGFR, mL/min/1.73m^2^ | Neg | -0.851 | <0.001 |
| Proteinuria, g/24 hour | Pos | 0.281 | <0.001 |
| MAP, mmHg | Pos | 0.228 | 0.001 |
| Hemoglobin, g/L | Neg | -0.184 | 0.007 |
| Serum albumin, g/L | Neg | -0.182 | 0.008 |
| Serum cholesterol, g/L | No Sig | 0.070 | 0.311 |
| Serum triglyceride, g/L | No Sig | 0.132 | 0.055 |
| LDL-cholesterol, g/L | No Sig | 0.063 | 0.359 |
| HDL-cholesterol, g/L | Neg | -0.163 | 0.017 |
| Serum IgA, g/L | Pos | 0.168 | 0.014 |
| Serum C3, g/L | No Sig | -0.002 | 0.979 |
| Serum uric acid, μmol/L | Pos | 0.479 | <0.001 |
| Serum erythropoietin, IU/L | Neg | -0.145 | 0.034 |
| Abbreviations: r, correlation coefficient; Pos, positive correlation; Neg, negative correlation; No Sig, no significant correlation; eGFR, estimated glomerular filtration rate; MAP, mean arterial blood pressure. | | | |

|  | **Cut Points** | **Remission failure %** | **Unadjusted OR**  **(95% Cl); *P*** | **Adjusted OR (95% Cl)** | | | |
| --- | --- | --- | --- | --- | --- | --- | --- |
|  |  |  |  | **Model 1^a^, *P*** | **Model 2^b^, *P*** | **Model 3^c^, *P*** | |
| Urinary β2-MG (μg/g Cr) | |  |  |  |  |  | |
| T1+T2 (n=111) | ≤ 459.31 | 14.4 | 1.0 (referent) | 1.0 (referent) | 1.0 (referent) | 1.0 (referent) | |
| T3 (n=55) | > 459.31 | 34.5 | 3.13 (1.45-6.75);  0.004 | 3.58 (1.57-8.19);  0.002 | 2.09 (0.81-5.40);  0.130 | 2.29 (0.87-6.03);  0.095 | |
| UTRF (mg/g Cr) |  |  |  |  |  |  | |
| T1+T2 (n=111) | ≤ 59.34 | 9.9 | 1.0 (referent) | 1.0 (referent) | 1.0 (referent) | 1.0 (referent) | |
| T3 (n=55) | > 59.34 | 43.6 | 7.04 (3.10-15.97);  <0.001 | 7.49 (3.22-17.43);  <0.001 | 3.30 (1.04-10.53);  0.043 | 3.43 (1.04-11.37);  0.044 | |
| Abbreviations: Cl, confidence interval; Cr, creatinine; OR, Odds ratio; BMI, body mass index; eGFR, estimated glomerular filtration rate; MAP, mean arterial blood pressure; MEST-C, histologic score based on mesangial hypercellularity, the presence of endocapillary proliferation, segmental glomerulosclerosis/adhesion, and severity of tubular atrophy/interstitial fibrosis, and crescents formation; T, tertile;  ^a^Model 1 adjusted for age, sex, MAP, BMI.  ^b^Model 2 adjusted for covariates in model 1 plus 24-hour proteinuria, eGFR and Oxford MEST-C score.  ^c^Model 3 adjusted for covariates in model 1 and 2 plus use of renin-angiotensin system inhibition and immunosuppression during follow-up. | | | | | | |  |

**Supplementary Table 4. Multivariable logistic analyses of urinary β2-MG and UTRF for predicting risk of IgAN progression**
